# Severe probable immune checkpoint inhibitor-related pneumonitis after thoracic radiotherapy complicated by acute pulmonary embolism in lung squamous cell carcinoma: a case report

**DOI:** 10.3389/fmed.2026.1860616

**Published:** 2026-06-17

**Authors:** Fangping Luo, Wanli Zhu, Yali Yu, Hongling Hu, Yingshan Cui, Haiqing Pan

**Affiliations:** Department of Radiotherapy, Jinhua Municipal Central Hospital, Jinhua, China

**Keywords:** high-flow nasal cannula, immune checkpoint inhibitor-related pneumonitis, lung squamous cell carcinoma, pulmonary embolism, thoracic radiotherapy

## Abstract

Immune checkpoint inhibitor-related pneumonitis (CIP) is a potentially life-threatening adverse event in lung cancer. We report a case of severe probable CIP complicated by acute pulmonary embolism (PE) in a 76-year-old man with stage IIIA lung squamous cell carcinoma after chemoimmunotherapy and thoracic radiotherapy. He developed progressive dyspnea and severe hypoxemia. Serial chest computed tomography (CT) showed bilateral interstitial and ground-glass abnormalities beyond the radiation field, favoring probable CIP, although superimposed infection and obstructive pneumonia could not be fully excluded. During hospitalization, CT pulmonary angiography confirmed acute PE, and lower-extremity ultrasound suggested calf venous thrombosis. The patient required high-flow nasal cannula and received methylprednisolone, tocilizumab, intravenous immunoglobulin, mycophenolate mofetil, antimicrobial therapy, and therapeutic anticoagulation. Despite partial clinical improvement, serial chest CT demonstrated continued radiographic progression. This case highlights the need for dynamic differential diagnosis, multidisciplinary management, and close bedside monitoring in complex post-radiotherapy CIP.

## 1 Introduction

Immune checkpoint inhibitors have become an important component of treatment for non-small cell lung cancer (NSCLC), but they can also cause immune-related adverse events involving multiple organs (1). Among these, immune checkpoint inhibitor-related pneumonitis (CIP) is relatively uncommon but clinically significant because severe cases may progress rapidly to hypoxemic respiratory failure and carry substantial mortality (2, 3). NSCLC appears to be a high-risk setting for CIP, and prior thoracic radiotherapy may further complicate both susceptibility and interpretation of subsequent pulmonary toxicity (4).

In clinical practice, the diagnosis of CIP is often challenging because its manifestations overlap with those of radiation pneumonitis, infectious pneumonia, and tumor-related obstructive inflammation (5). This problem is particularly pronounced in patients with thoracic malignancies who have received multimodal treatment and already have structural lung abnormalities (6). Current expert consensus emphasize that diagnosis and management rely on integration of treatment history, imaging distribution, exclusion of alternative causes, and repeated multidisciplinary reassessment rather than any single definitive test (7). Once severe disease develops, treatment usually requires prompt corticosteroid therapy, close monitoring of oxygenation, and escalation to additional immunomodulatory agents in selected steroid-refractory cases.

Although the clinical management of severe CIP has been increasingly discussed, reports remain limited when immune-mediated lung injury occurs in a post-radiotherapy lung and is further complicated by concurrent thromboembolic disease. Here, we report a case of severe probable CIP in a patient with stage IIIA lung squamous cell carcinoma after chemoimmunotherapy and thoracic radiotherapy, whose course was complicated by acute pulmonary embolism (PE), deep-venous thrombosis, and type I respiratory failure.

## 2 Case presentation

A 76-year-old man with stage IIIA (cT_2a_N_2a_M_0_) well-differentiated squamous cell carcinoma of the left lung was admitted on December 22, 2025, because of worsening chest tightness and dyspnea for 6 days. He had been diagnosed in April 2025 and subsequently received multimodal anti-cancer treatment, including albumin-bound paclitaxel plus carboplatin with toripalimab, several cycles of reduced-dose taxane, platinum, and fluorouracil chemotherapy combined with tislelizumab, computed tomography (CT)-guided intralesional chemotherapy ablation, intracavitary perfusion chemotherapy, bronchoscopic balloon dilation, and transarterial infusion therapy. From October 22 to November 26, 2025, the patient underwent thoracic radiotherapy (Figure 1). The prescribed dose was 54 Gy in 27 fractions to 95% of the planning target volume, and 26 fractions were actually delivered. The main lung dose parameters were V5 40%, V20 26%, V30 17%, and mean lung dose 12.82 Gy. After radiotherapy, the patient developed chest tightness and dyspnea, and radiative imaging at that time raised concern for either radiation pneumonitis or CIP. His symptoms partially improved after treatment with methylprednisolone and piperacillin-tazobactam, and he was discharged on a tapering course of oral methylprednisolone.

**Figure 1:**
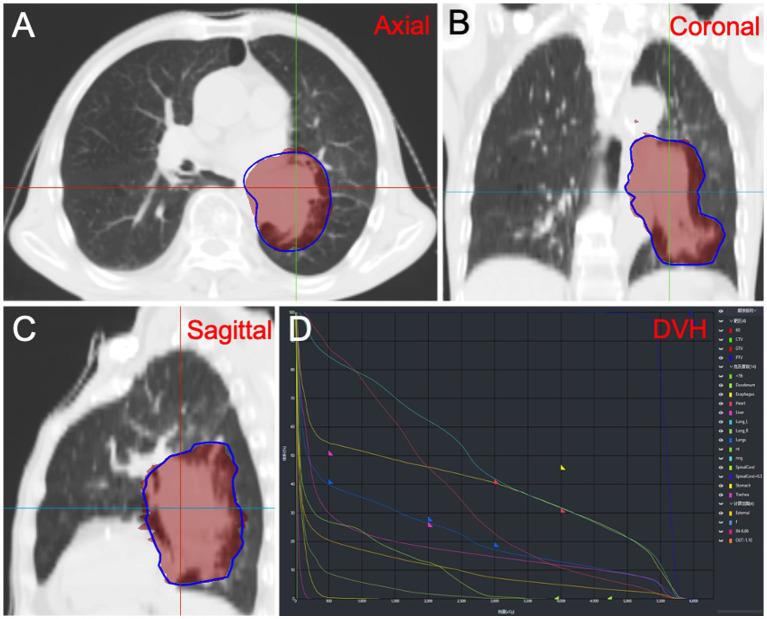
Thoracic radiotherapy planning images and dose-volume histogram. **(A–C)** Axial, coronal, and sagittal planning computed tomography images show the radiotherapy target region in the left lung, with target contours and dose distribution overlaid. **(D)** Dose-volume histogram (DVH) of the target volumes and organs at risk.

On December 17, 2025, his dyspnea recurred and worsened, particularly on exertion. Arterial blood gas analysis demonstrated marked hypoxemia with respiratory alkalosis (pH 7.547, PaCO_2_ 26.1 mmHg, PaO_2_ 52.9 mmHg). He received intravenous methylprednisolone and oral moxifloxacin before admission, but his symptoms persisted. On admission, the patient was alert, afebrile, and hemodynamically stable. Physical examination showed coarse breath sounds bilaterally and dry rales over the left lung, without peripheral edema. His body mass index was 18.7 kg/m^2^, and nutritional screening indicated increased nutritional risk.

During the early hospital course, the patient remained significantly dyspneic despite nasal cannula oxygen at 5 L/min. Repeat blood gas analysis continued to demonstrate hypoxemia. Chest CT scan on December 22, 2025, showed pulmonary infiltrates, together with left hilar/perihilar post-treatment changes and distal obstructive inflammatory changes in the left lung (Figure 2A). Multidisciplinary review subsequently favored probable CIP because the abnormalities evolved from unilateral to bilateral interstitial and ground-glass changes and, according to radiologic assessment, extended beyond the radiation field (Figure 1 and Figure 2). The main competing diagnoses were radiation pneumonitis, bacterial pneumonia, opportunistic infection, and tumor-related obstructive pneumonia. Therefore, the microbiological evaluation was broadened during hospitalization. Sputum and fungal culture were obtained, and the sputum culture yielded *Klebsiella aerogenes*. Antimicrobial therapy was subsequently adjusted according to susceptibility testing. *Candida albicans* was also detected in fungal culture, but this was interpreted cautiously in the clinical context and managed with oral care rather than regarded as definitive evidence of invasive fungal pneumonia. T-SPOT testing was negative. Bronchoscopy with bronchoalveolar lavage (BAL), including BAL-based multiplex pathogen testing, pneumocystis jirovecii testing, and shotgun metagenomic sequencing, were not performed because severe hypoxemia and respiratory instability made the procedure unsafe.

**Figure 2:**
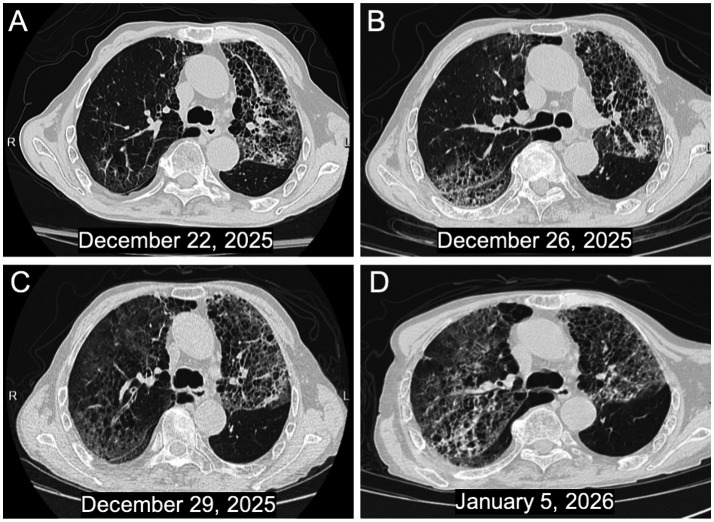
Serial chest computed tomography (CT) images demonstrating the progression of immune checkpoint inhibitor-related pneumonitis (CIP). **(A)** Initial CT shows left hilar/perihilar post-treatment changes with distal obstructive inflammatory changes in the left lung and new inflammatory abnormalities in the left upper lung. **(B–D)** Follow-up CT scans demonstrate progression from unilateral inflammatory changes to bilateral interstitial and ground-glass infiltrates, with distribution extending beyond the expected radiation field.

Serum biomarkers were interpreted as supportive but not diagnostic. Serial testing showed that C-reactive protein decreased from 17.48 mg/L on December 23, 2025, to 0.5 mg/L on December 31, 2025, and remained low thereafter, while procalcitonin remained low during hospitalization. In contrast, IL-6 was persistently elevated and increased to 1290.31 pg./mL by January 5, 2026. These findings did not exclude superimposed bacterial infection, particularly given the positive *Klebsiella aerogenes* culture and the left-sided obstructive inflammatory changes shown on CT, but they argued against uncontrolled bacterial sepsis as the sole explanation for the progressive bilateral interstitial abnormalities (Figures 2B–D). Accordingly, the pulmonary process was best characterized as severe probable CIP favored by multidisciplinary assessment, with concomitant bacterial infection and tumor-related obstructive pneumonia not fully excluded.

A major turning point occurred on December 24, 2025, when the patient’s D-dimer rose sharply to 16,994 μg/L. CT pulmonary angiogram (CTPA) (Figure 3) confirmed acute PE, although no radiographic evidence of right heart dysfunction or pulmonary infarction was seen. Subsequent echocardiography showed mild pulmonary hypertension, and lower-extremity vascular ultrasound suggested left calf intramuscular venous thrombosis. Troponin I was elevated during hospitalization, and cardiology consultation considered the myocardial injury most likely secondary to PE-related cardiopulmonary stress. Therapeutic anticoagulation with enoxaparin was therefore initiated after contraindications were excluded.

**Figure 3:**
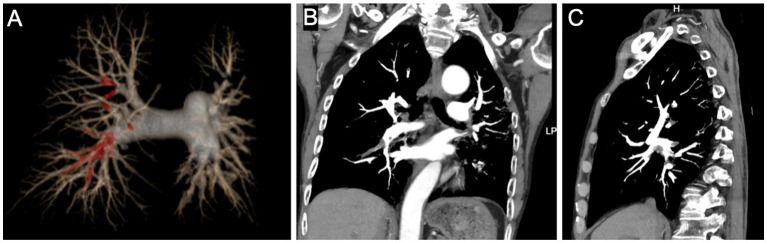
Computed tomography pulmonary angiography (CTPA) confirming acute pulmonary embolism (PE). **(A)** Three-dimensional reconstruction image provides an overview of the pulmonary vasculature. **(B, C)** Coronal and sagittal CTPA images clearly demonstrate a filling defect within a segmental branch of the right pulmonary artery, which is diagnostic of acute PE.

The patient developed type I respiratory failure and required stepwise escalation of oxygen therapy, as summarized in Table 1. Oxygen support progressed from nasal cannula oxygen to reservoir mask oxygen and then to high-flow nasal cannula (HFNC). HFNC was initiated on December 25, 2025, at 40 L/min. After a temporary trial of face-mask oxygen at 7 L/min on December 29–30, 2025, HFNC was resumed on December 31, 2025, because oxygen dependence and exertional dyspnea persisted. From December 31, 2025, to January 6, 2026, HFNC was maintained at 40 L/min. On January 7, 2026, the flow rate was increased to 45 L/min. The lowest recorded peripheral oxygen saturation (SpO₂) during HFNC support was 90% on January 5, 2026, accompanied by worsening dyspnea with a visual analog scale (VAS) score of 7. According to CTCAE criteria (8), the pneumonitis was classified as grade 3 because the patient had severe respiratory symptoms, required hospitalization and continuous oxygen therapy, and needed prolonged HFNC support. Non-invasive ventilation or intubation was not used because oxygenation could still be maintained with HFNC under close monitoring. No intensive care unit admission was documented during hospitalization. During this period, bedside supportive care focused on continuous monitoring of oxygen saturation, respiratory effort, HFNC tolerance, and arterial blood gas trends. After anticoagulation was started, close surveillance for hemoptysis and other bleeding manifestations was maintained, together with oral care, secretion management, and infection-prevention measures during intensified immunosuppression. Serum sodium, liver function, and overall treatment tolerance were also monitored to support early multidisciplinary intervention when complications emerged.

**Table 1:** Progression of oxygen therapy and HFNC support during hospitalization.

Date	Respiratory support	Oxygen flow rate	SpO₂	Dyspnea VAS
Dec 22, 2025	Nasal cannula	5 L/min	79%	5
Dec 23, 2025	Nasal cannula	5 L/min	91%	5
Dec 24, 2025	Reservoir mask	8 L/min	93%	6
Dec 25–28, 2025	HFNC	40 L/min	98%	6
Dec 29–30, 2025	Face-mask	7 L/min	97%	6
Dec 31, 2025-Jan 1, 2026	HFNC	40 L/min	98%	6
Jan 2–3, 2026	HFNC	40 L/min	96%	6
Jan 4, 2026	HFNC	40 L/min	95%	6
Jan 5, 2026	HFNC	40 L/min	90%	7
Jan 6, 2026	HFNC	40 L/min	93%	7
Jan 7, 2026	HFNC	45 L/min	93%	7

Because severe hypoxemia and radiographic abnormalities persisted after more than 72 h of systemic corticosteroid therapy, the clinical course was considered poorly corticosteroid-responsive probable CIP, or clinically steroid-refractory probable CIP. This interpretation was made cautiously because acute PE, *Klebsiella aerogenes* infection, and tumor-related obstructive pneumonia may also have contributed to persistent hypoxemia. Immunomodulatory therapy was escalated under multidisciplinary guidance. Methylprednisolone was increased to 100 mg/day, followed by tocilizumab 400 mg on December 26, intravenous immunoglobulin 20 g daily for 3 days starting on December 30, and mycophenolate mofetil 1 g twice daily from January 1, 2026. Tocilizumab was selected because IL-6 was markedly elevated and rheumatology consultation supported IL-6 blockade, whereas infliximab was considered less favorable in the setting of possible superimposed infection. Antimicrobial therapy was adjusted according to microbiologic findings, with piperacillin-tazobactam replaced by cefoperazone-sulbactam after *Klebsiella aerogenes* was reported to be resistant to the former. Trimethoprim-sulfamethoxazole was also administered during intensified immunosuppression.

By early January 2026, the patient’s dyspnea at rest had partially improved, and inflammatory markers and D-dimer levels had decreased from peak values, but exertional dyspnea persisted. Serial chest CT scans nevertheless demonstrated continued radiographic progression rather than interval improvement (Figure 2). A chronological summary of the patient’s cancer treatment, immunotherapy exposure, radiotherapy, symptom progression, imaging evolution, PE diagnosis, respiratory support, and immunomodulatory escalation was shown in Figure 4.

**Figure 4:**
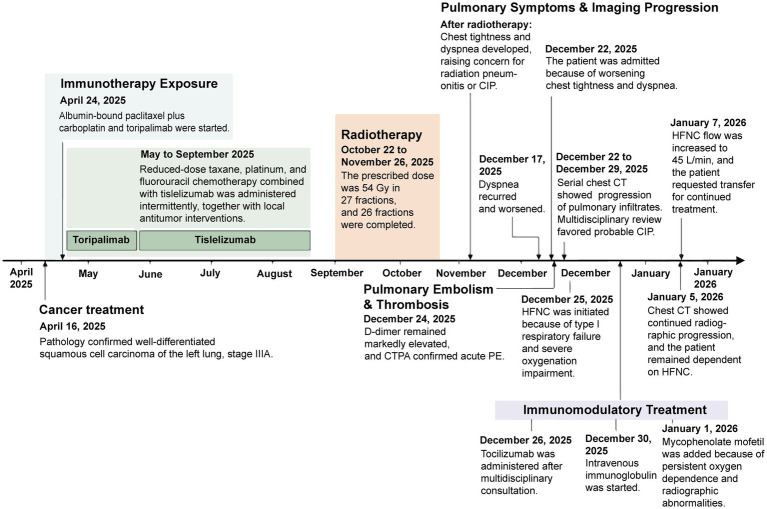
Chronological timeline of the patient’s clinical course. The timeline summarizes key events from cancer diagnosis and antitumor treatment to pulmonary deterioration, pulmonary embolism diagnosis, high-flow nasal cannula support, and immunomodulatory escalation.

## 3 Discussion

Severe CIP is uncommon but potentially fatal, particularly in patients with NSCLC treated with PD-1 inhibitors (9, 10). The main significance of the present case lies not only in the severity of respiratory failure, but also in the diagnostic complexity created by combined chemoimmunotherapy, recent thoracic radiotherapy, possible superimposed infection, and tumor-related obstructive inflammation. The leading diagnosis was supported by the temporal relationship to PD-1 inhibitor exposure, progressive hypoxemic respiratory failure, bilateral interstitial and ground-glass abnormalities extending beyond the radiation field. However, diagnostic certainty was limited by the inability to perform bronchoscopy and BAL, the absence of BAL-based pneumocystis jirovecii testing or metagenomic sequencing, and the positive sputum culture for *Klebsiella aerogenes*. Thus, the diagnosis in this case was probabilistic and clinicoradiologic, rather than pathologically or microbiologically definitive. In real-world thoracic oncology practice, this distinction is important because severe CIP often has to be treated urgently while infection and other pulmonary causes are being investigated in parallel (7, 11).

Radiologic differentiation between radiation pneumonitis and CIP was central to the diagnostic reasoning in this case. The timing of symptom worsening within several weeks after thoracic radiotherapy was compatible with radiation pneumonitis, and therefore did not by itself establish CIP. However, the distribution and evolution of the abnormalities were less typical of isolated radiation pneumonitis. Radiation pneumonitis usually conforms to the irradiated volume or shows a geographic relationship with the treatment field, whereas this patient’s abnormalities evolved from left-sided inflammatory changes to multifocal bilateral interstitial and ground-glass infiltrates, with lesions extending beyond the expected radiation field and later progression in the contralateral lung (12). The involvement was bilateral but not completely symmetrical. The overall distribution was non-geographic rather than confined to the high-dose region. The predominant CT pattern was an interstitial and ground-glass inflammatory pattern with organizing pneumonia-like features, while diffuse alveolar damage could not be confirmed because tissue sampling was not feasible. The radiotherapy plan and dose-volume histogram were reviewed together with the serial diagnostic CT images, and the extent of the later bilateral infiltrates was considered discordant with isolated in-field radiation pneumonitis.

Comparison with previously published cases helps place our findings in context. Similar to the cases reported by Zhu et al. (13) and Yin et al. (14), our patient developed progressive inflammatory lung injury after PD-1 inhibitor exposure, with severe hypoxemia and the need for intensive respiratory support. However, our case differed in two important respects. First, prior thoracic radiotherapy substantially complicated the differential diagnosis, because the clinical timing overlapped with possible radiation pneumonitis, whereas the bilateral, non-geographic, extra-field CT progression favored probable CIP over isolated radiation-related injury (15). Second, unlike more diagnostically straightforward immune-mediated cases, our patient had persistent left obstructive inflammatory changes and a positive sputum culture for *Klebsiella aerogenes*. This finding was clinically relevant and justified targeted antimicrobial therapy, but it did not fully explain the progressive bilateral interstitial and ground-glass abnormalities. Therefore, bacterial infection was considered a contributor rather than a complete alternative explanation.

Another distinctive aspect of this case was the coexistence of acute PE and calf venous thrombosis. The thromboembolic event was likely multifactorial. First, active lung cancer itself is a well-recognized prothrombotic condition. Second, reduced mobility during progressive dyspnea and hospitalization may have further increased venous stasis. Third, systemic inflammation related to severe pulmonary injury, infection risk, and immune activation may have promoted endothelial dysfunction and hypercoagulability. Finally, immune checkpoint inhibitor therapy may also have contributed to venous thromboembolism (VTE) risk, although a causal relationship cannot be established in a single case. Recent reviews have suggested that immune checkpoint inhibitor-associated inflammation may contribute to thrombosis, but reported VTE rates vary across clinical settings and study designs (16). Therefore, PE in this patient should be interpreted as a multifactorial complication rather than an event attributable to CIP alone.

Clinically, the diagnosis of PE changed the interpretation and management of respiratory failure. The patient’s hypoxemia reflected both parenchymal lung injury from probable CIP and vascular obstruction from acute PE. This mixed mechanism required simultaneous immunosuppressive treatment for probable CIP, therapeutic anticoagulation for PE and calf venous thrombosis, and close monitoring for bleeding, infection, and respiratory deterioration. The coexistence of PE may also help explain why clinical improvement was incomplete despite aggressive anti-inflammatory therapy.

Our patient also resembled previously reported cases of corticosteroid-refractory or recurrent CIP in that radiographic progression continued despite corticosteroid therapy, necessitating escalation to tocilizumab, intravenous immunoglobulin, and mycophenolate mofetil (17, 18). Yet even with this stepwise intensification, the response was incomplete. Bedside symptoms at rest and selected laboratory parameters improved partially, whereas serial CT scans still showed continued progression. This discordance is clinically instructive. It suggests that in severe probable CIP, especially when modified by thrombosis and infection risk, short-term clinical stabilization should not be over interpreted as disease control. Response assessment should remain multidimensional, incorporating symptoms, oxygen requirement, imaging evolution, and treatment tolerance rather than relying on any single marker (19).

Although this report is clinically oriented, it also highlights the importance of nursing as an integral part of complex management rather than a separate supportive narrative. Existing literature has emphasized the bedside role of nurses in monitoring oxygenation, detecting treatment-related adverse events, and maintaining patient safety during immunotherapy-associated complications (20, 21). In our case, these functions were especially relevant during HFNC support, anticoagulation, and intensified immunosuppression, when continuous observation of respiratory effort, bleeding manifestations, secretion burden, and metabolic abnormalities directly informed multidisciplinary decision-making.

The limitations of this case should also be acknowledged. Broncho alveolar lavage was not performed, microbiologic exclusion was incomplete, and long-term outcome was unavailable. Nevertheless, when viewed alongside the published literature, this case remains clinically informative because it demonstrates how severe probable CIP may evolve in a post-radiotherapy lung and how its management becomes substantially more difficult when complicated by concurrent PE and persistent radiographic progression.

## 4 Conclusion

In conclusion, this case demonstrates that severe probable CIP after chemo immunotherapy and thoracic radiotherapy may evolve as a complex, multi-causal respiratory syndrome rather than an isolated immune toxicity. When complicated by PE, venous thrombosis, infection risk, and persistent radiographic progression, effective management depends not only on timely immunomodulatory escalation, but also on continuous bedside assessment, supportive care, and coordinated multidisciplinary decision-making.

## Data Availability

The original contributions presented in the study are included in the article/supplementary material, further inquiries can be directed to the corresponding author.

## References

[1] Mohammed N Xiao EH Mohsen S Xiong Z Zhou R. PD-1/PD-L1 inhibitor treatment and its impact on clinical imaging in non-small cell lung cancer: a systematic review and meta-analysis of immune-related adverse events. Front Oncol. (2023) 13:1191681. doi: 10.3389/fonc.2023.1191681, 37841435 PMC10571717

[2] Zhou P Zhao X Wang G. Risk factors for immune checkpoint inhibitor-related pneumonitis in Cancer patients: a systemic review and Meta-analysis. Respiration. (2022) 101:1035–50. doi: 10.1159/000526141, 36108598

[3] Lin GF Xu Y Lin H Yang DY Chen L Huang LL . The association between the incidence risk of pneumonitis and PD-1/PD-L1 inhibitors in advanced NSCLC: a meta-analysis of randomized controlled trials. Int Immunopharmacol. (2021) 99:108011. doi: 10.1016/j.intimp.2021.108011, 34426108

[4] Zhou C Li M Wang Z An D Li B. Adverse events of immunotherapy in non-small cell lung cancer: a systematic review and network meta-analysis. Int Immunopharmacol. (2022) 102:108353. doi: 10.1016/j.intimp.2021.108353, 34883352

[5] Cheng J Pan Y Huang W Huang K Cui Y Hong W . Differentiation between immune checkpoint inhibitor-related and radiation pneumonitis in lung cancer by CT radiomics and machine learning. Med Phys. (2022) 49:1547–58. doi: 10.1002/mp.15451, 35026041 PMC9306809

[6] Peiliang Wang MD Yikun Li MM Mengyu Zhao MM Jinming Yu MD Feifei Teng MD. Distinguishing immune checkpoint inhibitor-related pneumonitis from radiation pneumonitis by CT radiomics features in non-small cell lung cancer. Int Immunopharmacol. (2024) 128:111489. doi: 10.1016/j.intimp.2024.111489, 38266450

[7] Wang W Wang Q Xu C Li Z Song Z Zhang Y . Chinese expert consensus on the multidisciplinary management of pneumonitis associated with immune checkpoint inhibitor. Thorac Cancer. (2022) 13:3420–30. doi: 10.1111/1759-7714.14693, 36268845 PMC9715776

[8] Schneider BJ Naidoo J Santomasso BD Lacchetti C Adkins S Anadkat M . Management of Immune-Related Adverse Events in patients treated with immune checkpoint inhibitor therapy: ASCO guideline update. J Clin Oncol. (2021) 39:4073–126. doi: 10.1200/JCO.21.01440, 34724392

[9] Tone M Izumo T Awano N Kuse N Inomata M Jo T . High mortality and poor treatment efficacy of immune checkpoint inhibitors in patients with severe grade checkpoint inhibitor pneumonitis in non-small cell lung cancer. Thorac Cancer. (2019) 10:2006–12. doi: 10.1111/1759-7714.13187, 31482678 PMC6775002

[10] Zhang Q Tang L Zhou Y He W Li W. Immune checkpoint inhibitor-associated pneumonitis in non-small cell lung Cancer: current understanding in characteristics, diagnosis, and management. Front Immunol. (2021) 12:663986. doi: 10.3389/fimmu.2021.663986, 34122422 PMC8195248

[11] Li S Geng Z Hong S Zhang J Yang Y Wei Q . Advances in the mechanisms, imaging characteristics and management strategies for immune checkpoint inhibitor-related pneumonitis. Front Immunol. (2025) 16:1656063. doi: 10.3389/fimmu.2025.1656063, 40963607 PMC12436323

[12] Chen X Sheikh K Nakajima E Lin CT Lee J Hu C . Radiation versus immune checkpoint inhibitor associated pneumonitis: distinct radiologic morphologies. Oncologist. (2021) 26:e1822–32. doi: 10.1002/onco.13900, 34251728 PMC8488797

[13] Zhu Y Yu J Ren Q Wu X Xu H Tian T . Clinical characteristics and biomarkers of severe immune checkpoint inhibitor-related pneumonitis triggered by immunotherapy followed by radiation: a case report. Front Immunol. (2024) 15:1454114. doi: 10.3389/fimmu.2024.1454114, 39650655 PMC11621207

[14] Yin B Xiao J Li J Liu X Wang J. Immune-related organizing pneumonitis in non-small cell lung cancer receiving PD-1 inhibitor treatment: a case report and literature review. J Cancer Res Ther. (2020) 16:1555–9. doi: 10.4103/jcrt.JCRT_971_20, 33565499

[15] Noda-Narita S Naito T Udagawa H Goto K Miyawaki T Mamesaya N . Nivolumab-induced radiation recall pneumonitis in non-small-cell lung cancer patients with thoracic radiation therapy. Cancer Sci. (2023) 114:630–9. doi: 10.1111/cas.15621, 36285515 PMC9899626

[16] Wang TF Carrier M. Immune Checkpoint Inhibitors-Associated Thrombosis: Incidence, Risk Factors and Management. Curr Oncol. (2023) 30:3032–46. doi: 10.3390/curroncol30030230, 36975443 PMC10047296

[17] Gatti-Mays M Gulley JL. Real-world insights on preferred treatments for steroid-refractory immune checkpoint inhibitor-induced pneumonitis. J Immunother Cancer. (2021) 9:e002252. doi: 10.1136/jitc-2020-002252, 33602697 PMC7896600

[18] Yu J Liu X Ma X Liang L Liu Y Yin W . Recurrent checkpoint inhibitor-related pneumonitis refractory to corticosteroid treatment: a case report and literature review. Clin Pharmacol. (2025) 17:235–42. doi: 10.2147/CPAA.S534323, 40861846 PMC12374699

[19] Xu Y Chen R Pan R Gao X Huang H Wang M. Clinical management of checkpoint inhibitor pneumonitis: focus, challenges, and future directions. Chin Med J Pulm Crit Care Med. (2025) 3:29–40. doi: 10.1016/j.pccm.2024.12.001, 40226598 PMC11993061

[20] Lewis C. Programmed Death-1 inhibition in Cancer with a focus on non-small cell lung Cancer: rationale, nursing implications, and patient management strategies. Clin J Oncol Nurs. (2016) 20:319–26. doi: 10.1188/16.CJON.319-326, 27206299

[21] Sun YY Wei LM Qian Y. The role of nursing in enhancing quality of life for lung cancer patients receiving targeted and immunotherapy: challenges, opportunities, and future directions. Hum Vaccin Immunother. (2025) 21:2506302. doi: 10.1080/21645515.2025.2506302, 40390558 PMC12101592

